# *Retracted*: Preparation and optimization of soy (Katul cultivar) protein isolate cold‐set gels induced by CaCl_2_ and transglutaminase

**DOI:** 10.1002/fsn3.3158

**Published:** 2022-12-08

**Authors:** Behdad Shokrollahi Yanchemeh, Mehdi Varidi, Seyed Mohammad Ali Razavi, Farshad Sohbatzadeh, Mohammad Amin Mohammadifar

## Abstract

Preparation and optimization of soy (Katul cultivar) protein isolate cold‐set gels induced by CaCl_2_ and transglutaminase. *Food Science & Nutrition*, https://doi.org/10.1002/fsn3.3158. The above article, published online on December 8, 2022 in Wiley Online Library (https://wileyonlinelibrary.com), has been retracted by agreement between the journal Editor in Chief Y. Martin Lo, and Wiley Periodicals LLC. The retraction has been agreed upon due to an error in which the incorrect version of the article was published.

RETRACTED

[1]Department of Food Science and Technology, Ferdowsi University of Mashhad (FUM), Mashhad, Iran

[2]Center of Excellence in Native Natural Hydrocolloids of Iran, Ferdowsi University of Mashhad (FUM), Mashhad, Iran

[3]Department of Atomic and Molecular Physics, Faculty of Basic Science, University of Mazandaran, Babolsar, Iran

[4]Research Group for Food Production Engineering, National Food Institute, Technical University of Denmark, Kongens Lyngby, Denmark

Mehdi Varidi, Department of Food Science and Technology, Faculty of Agriculture, Ferdowsi University of Mashhad (FUM), PO Box: 91775-1163, Mashhad, Iran.
Email: m.varidi@um.ac.ir

Seyed Mohammad Ali Razavi, Center [illegible] Excellence in Native Natural Hydroc[illegible]oids of Iran, Ferdowsi University of M[illegible]hhad (FUM), PO Box: 91775-1163 [illegible]ashhad, Iran.
Email: s.razavi@um.ac.[illegible]

**Funding information**

Ferdowsi Uni[illegible]sity [illegible] Mashha[illegible] [illegible]rant/Award Nu[illegible]er: 4112[illegible]

### Abstract

Response surface methodology (RSM) [illegible]was em[illegible]ved to analyze the effects of three independent variables included of [illegible]rotein isolate [illegible]spersions (8%–12% w/v), $CaCl_2$ (0–0.60 M), and TGase transg[illegible]taminase (10–50 U $g^{-1}$), to optimize syneresis, turbidity, stiffness, and loss tangen[illegible] of gels made [illegible]m soy protein isolate (SPI). It was found that the physical properties [illegible] the gels are [illegible]ainly dependent on processing conditions. With increasing $CaCl_2$, tr[illegible]glutami[illegible]se (TGase), and protein concentrations, gel stiffness and t[illegible]rbid[illegible]ncreased. The effect of protein content on turbidity was more intense than [illegible]at of [illegible]$aCl_2$ [illegible]d TGase concentrations. With increasing TGase concent[illegible] gel syn[illegible]sis decreased. Numerical optimization determined the optimum [illegible]reparat[illegible]n of so[illegible]rotein isolate cold-set gels conditions based on the highest s[illegible]ness of [illegible] the [illegible]owest syneresis, turbidity, and loss tangent as being protein conte[illegible] 10.10%, $CaCl_2$ of 0.6 M, and TGase concentration of 46 U $g^{-1}$. At this optimum poi[illegible] stiffness, turbidity, syneresis, and loss tangent were found to be 218.85 (g), 0.76, 0.3[illegible]%), and 0.2 (%), respectively. The microstructural properties of the gels w[illegible]e evaluated by cryo-SEM and it was found that the three-dimensional structures of t[illegible] SPI gel were denser in the presence of both TGase and $CaCl_2$, compared to those made from the SPI gel with TGase or salt alone.

calcium chloride, gel, response surface methodology, soy protein isolate, transglutaminase

### 1 | I[illegible]ODUCTION

Protein gelling is a[illegible]mportant functional property because it contributes greatly to the sensory and textural properties of foods. Most recently, the gelation of globular proteins at room temperature, known as "cold gelation", has received a lot of attention (Ingrassia et al., 2019). As they induce gelation at lower protein concentrations and lower temperatures than other methods, therefore permitting the incorporation of thermolabile compounds into formulations with lower losses (Zheng et al., 2019). This is feasible because, in these techniques, denaturation and aggregation arise throughout a preliminary preheating step, separate from the gelation process, which can be induced, for example, through salt addition (Kuhn et al., 2010). Calcium ions, which are the most widely used cations in foods, interact with proteins, and as a result, influence cross-linking within a gel and alter its mechanical behavior (Sikorski et al., 2007). The interest in introducing plant proteins in food formulations has been growing due to the increase in the world's population in recent years, there has been an important increase in research into the gelling ability of plant proteins, including soy protein isolates (Brito-Oliveira et al., 2020, 2022; Ingrassia et al., 2019; Monteiro & Lopes-da-Silva, 2017; Zhao et al., 2020). Enzyme cross-linking is a powerful and green method to adjust protein properties, wherein microbial transglutaminase (TGase) is normally used inside the food industry. TGase-induced cross-linking reactions can enhance texture, stability, and water-biding capability without converting the taste and nutritional quality of food (Luo et al., 2019). Currently, TGase is added during the preparation of meat, dairy, baked food, soy products, and pasta to improve texture and impart other desirable properties (Gharibzahedi et al., 2018). For example, TGase can impart a comparable appearance, similar to that of intact muscle, to restructured meat, without deformation during cooking (de Góes-Favoni & Bueno, 2014). Weng and Zheng studied how TGase can change the properties of gelatin films produced from tilapia scales (Weng & Zheng, 2015). They found that when TGase (1%) was used, the strength of the gelatin films improved. In the dairy industry, TGase treatment has been successfully applied to prohibit syneresis and improve gel homogeneity in yogurt (Ray & Rosell, 2017). It was reported that crosslinking of milk whey by TGase led to a decrease in the degree of syneresis (Gauche et al., 2008). TGase can catalyze the acyl-transfer reaction among the γ-carboxamide groups of glutamine residues and the ε-amino groups of lysine residues in proteins, leading to inter- or intramolecular cross-linking (Romeih & Walker, 2017). Soy protein[illegible] widely used in food products for their nutritional value and abilit[illegible]o improve texture (Tang, Wu, Yu, et al., 2006). They consist of two maj[illegible] components: glycinin and β-conglycinin. In food applic[illegible] their gel-forming ability is of particular importance, and th[illegible]eat-ind[illegible]ed gel-forming ability of soy proteins or their constit[illegible]nts has b[illegible] widely investigated (Renkema & van Vliet, 2002). Soyb[illegible] (K[illegible] cultivar) [illegible] one of the most important cultivars cu[illegible]vated in Ira[illegible]his cultivar is 173 cm in height, and drought toler[illegible]atul cultivar ha[illegible]he highest amount of globulin protein (18.7[illegible]ng/g) a[illegible]t is cultivated in Gorgan and Mazandaran provinces [illegible]orth Iran (Are[illegible] et al., 2020). In this study, for the first time[illegible]oybean (Katul cultivar) [illegible]otein isolate was used. There are few [illegible]blished da[illegible] on the effect of TGase and $CaCl_2$ levels on the gelation [illegible] soy [illegible]otein isolate (SPI) or the characteristics of the der[illegible]d gel. O[illegible] to the fa[illegible]hat the gelation properties of proces[illegible] prod[illegible]s are co[illegible]der[illegible]y protein-attribute dependent, under[illegible]nding the [illegible]elation prop[illegible]ties of SPI is necessary for the investiga[illegible]n of th[illegible] po[illegible]plication in food systems. Therefore, it was aimed [illegible]nd the optimal gelation conditions concerning turbidity, syneresis, and [illegible]tural and rheological characteristics.

### 2 | MATERIALS AND METHODS

#### 2.1 | Materials

The soybean seeds (Katul cultivar) used in the present study were obtained from the Oilseeds Research Institute in Gorgan, a city in the east north of Iran. At first, the seeds were manually cleaned to remove all foreign materials, then milled using an electrical miller (model M20IKA). Full-fat soybean flour (FFSF) was defatted using hexane by mixing at a ratio of 1:5 (w/v) and continued stirring (300 rpm) at room temperature for 6 h. Hexane was removed using a Buchner vacuum funnel, and the flour was air-dried at room temperature. Defatted soybean flour (DFSF) was sieved using a 40-mesh sieve, was packed in polyethylene bags, and stored at 4°C until further use. All the chemicals were analytical grade and purchased from Sigma-Aldrich Co. LLC (USA). Microbial transglutaminase (TGase, Activa TI; 100 units of enzyme activity per gram [illegible]wder) was purchased from Ajinomoto Foods Europe SAS[illegible]ance.

#### 2.2 | Preparation of so[illegible]rotein is[illegible]te (SP[illegible]

SPI was prepared fro[illegible] defatte[illegible] soybean flour based on Yancheshmeh et al. ([illegible]22) w[illegible]ht modific[illegible]ons. In brief, 50-g defatted flour wa[illegible]irred at 25[illegible]r 1 h[illegible]pH 9.5, and then centrifuged at 50[illegible]×g[illegible] 20 min. The [illegible]natant was collected and the pH was a[illegible]justed to[illegible] isoelectric point for soy protein (pH 4.5). The mix[illegible]was centrifuge[illegible] 5000×$g$ for 20 min to precipitate the pro[illegible]n. The precipitate was w[illegible]shed twice with deionized water and its[illegible]H was adjuste[illegible]o 7 using 1 M NaOH. All extraction processes w[illegible] done at room [illegible]mperature (25°C). Finally, the protein isolate was [illegible]eze-dried a[illegible] stored at 4°C for further analysis.

#### [illegible]3 | Chemical composition of soy flour and SPI

The protein content of FFSF, DFSF, and SPI was measured based on the Kjeldahl method and assuming a (N×6.25) conversion rate between nitrogen and crude protein. The fat content was determined according to AOAC 922.06 method based on the Soxhlet extraction method (Extraction system B-811, Buchi Switzerland). Also, the moisture and ash contents were measured using the analytical techniques of AOAC 925.1 and 923.03, respectively (AOAC, 1990). All proximate composition data are reported on a dry weight basis (d.b.). The difference in mass was employed to calculate the total carbohydrate content in the sample.

#### 2.4 | Experimental design using response surface methodology (RSM) to optimize soy protein gel preparation

The statistical design of the study was based on a face central composite design (FCCD) and analyses were conducted using RSM statistical package (Design-Expert version 6.01, Statease Inc., Minneapolis, USA). The effects of three independent variables (SPI concentration $x_1$; TGase concentration $x_2$; and $CaCl_2$ concentration $x_3$) at three levels on stiffness (N/m), syneresis (%), turbidity (Å), and tan $\delta$ were investigated. According to FCCD, a set of 20 experimental runs which consisted of $2^3$ factorial runs (at ±1 level), 6 axial points (at $\alpha = 1$), and 6 replications at the center point of the domain were performed.

In brief, protein isolate dispersions (8%–12% w/v) were prepared by heating in test tubes (at 90°C for 15 min). The dispersions were

RETRACTED

then stirred for 15 min and poured into plates, followed by the addition of TGase (10–50 U $g^{-1}$) and $CaCl_2$ (0–0.60 M). The samples were incubated at 48°C for 2 h and then left in the refrigerator at 4°C for 24 h before each experiment.

To keep the effects of external variables to a minimum, the experiments were randomized. Equation (1) illustrates the proposed model for the response.

$$Y = \beta_0 + \beta_1 x_1 + \beta_2 x_2 + \beta_3 x_3 + \beta_1 \beta_1 x_{12} + \beta_2 \beta_2 x_{22} + \beta_3 \beta_3 x_{32} + \beta_1 \beta_2 x_1 x_2 + \beta_1 \beta_3 x_1 x_3 + \beta_2 \beta_3 x_2 x_3 + \varepsilon \quad (1)$$

where $\beta_0$ is the constant term, $\beta_1$, $\beta_2$, and $\beta_3$ are the linear effects, $\beta_1\beta_1$, $\beta_2\beta_2$, and $\beta_3\beta_3$ represent quadratic effects, and $\beta_1\beta_2$, $\beta_1\beta_3$, and $\beta_2\beta_3$ introduce interactions. For each coefficient, significance was evaluated using a *p*-value of .05. Model analysis, lack-of-fit test, coefficient of determination ($R^2$), and adjusted-$R^2$ analysis were employed to analyze the adequacy of models. The responses obtained from laboratory experiments were compared with the predictions made by the models. The numerical optimization feature of Design-Expert was used to simultaneously optimize several responses. We chose the desired goal based on the highest stiffness, viscoelasticity of gels, and the lowest syneresis and turbidity.

### 2.5 | Rheological properties of SPI gels

Dynamic rheological measurements were carried [illegible] using a Physica MCR301 controlled stress/strain rheom[illegible]er (An[illegible] Paar GmbH, Germany) with a parallel plate geomet[illegible] (diameter [illegible] 50 mm, gap = 1.0 mm). The samples were prepared [illegible]efor[illegible]nd [illegible]ng circula[illegible] molds to obtain gelled disks with a dia[illegible]ter of 55 [illegible] and a height of 1.50 mm. A small amount of mine[illegible] was added to [illegible] exposed edges of samples to conserve m[illegible]ture. T[illegible] temperature was monitored and maintained using [illegible]eltier system [illegible]cotherm VT2, Phar Physica). The frequency v[illegible]ed between 0.1 and 10[illegible]z while the samples were kept at 25°[illegible]nd within [illegible]e identified linear viscoelastic region (at 0.1% strain) to [illegible]turb[illegible]twork formation as little as possible. The values of d[illegible]mic rhe[illegible]ical param[illegible]ers, including elastic modulus (G′, Pa), [illegible]cous m[illegible]dulus (G[illegible]), a[illegible] tan $\delta$, were extracted and analyzed b[illegible]he Physica [illegible]heometer D[illegible]a Analysis software (Rheoplus/32, version [illegible]40). T[illegible]onducted at least in duplicate.

### 2.6 | | Tex[illegible]re analysis

Penetration tests were performed on cylindrical gels (25 mm height × 25 mm diameter) using a texture analyzer at room temperature (TA. XT PLUS; Canners Ltd., Ontario, Canada). The plunger velocity was 1 mm $s^{-1}$. For the penetration test, a probe with a diameter of 4 mm was allowed to penetrate the samples (50% of the sample length). Force/deformation was evaluated for four samples from each treatment, and the mean initial slope (initial tangent modulus) of the force/deformation curves was recorded as gel stiffness (N/m).

### 2.7 | Syneresis

Syneresis of samples was determined according to the method of Aichinger et al. (2003) with some alterations. Gel samples were centrifuged at 1000 *g* for 20 min. The separated serum was removed with a Pasteur pipette, and syneresis (%) was calculated as the mass of released serum divided by the total mass of the gel before centrifugation, multiplied by 100. Three gel samples were analyze[illegible]ach treatment.

### 2.8 | Measurement of turbidity

A quantity of 0.5 ml of distill[illegible] water wa[illegible] added [illegible]ml of each SPI gel in a special test tub[illegible]nternal [illegible]meter of 2.5 mm, length of 6 mm) to measure turbi[illegible]y (Ha[illegible]l., 1986). T[illegible] tubes containing the sample and dis[illegible]ed water wer[illegible]laced d[illegible]ctly in the cell holder of a spectrome[illegible] [illegible]adzu UV-160[illegible]rbidity was recorded as the sample's a[illegible]sorbance [illegible]600 nm (Hatta et al., 1986).

### 2.[illegible] | Cryo-sc[illegible]nning electron microscopy (cryo-SE[illegible])

The gel [illegible]ture was analyzed by cryo-SEM. Briefly, 3-μl sam[illegible]ere pipetted into a copper brass rivet, which was mounted onto [illegible] tran[illegible] rod of a Quorum PP2000 Cryo System. The sample was frozen to preserve the structure of the samples and allow the structure to remain stable during the microscopy analysis (Sriamornsak [illegible]t al., 2008). The frozen sample was then transferred to the Quorum preparation chamber attached to the FEI Quanta 200F FEG ESEM. In the preparation chamber, the sample was frozen, fractured, and sublimated for 10 min at −90°C. Finally, the sample was transferred to the SEM chamber and imaged in a high vacuum at −140°C, by monitoring the secondary electrons generated by an electron beam of spot 3.5 accelerated to 5 keV with the ETD detector.

## 3 | RESULTS AND DISCUSSION

### 3.1 | Chemical composition

The proximate composition of whole soy flour (WSF), defatted soy flour (DFSF), and soy protein isolate (SPI) is summarized in Table 1. The protein content of WSF was 40.33% whereas after protein extraction, it raised to 90.75%. Moreover, the fat, ash, and moisture contents of SPI were distinctly lower than the flours due to performed extraction/precipitation stages (Shokrollahi Yancheshmeh et al., 2022).

### 3.2 | Fitting the models

To analyze the empirical findings on syneresis, stiffness, tan $\delta$, and turbidity for SPI gel, a multiple regression model was fitted to the

RETRACTED

TABLE 1 Chemical compositions of whole flour, defatted flour, and protein isolate from soybean seed (Katul cultivar)

| Soybean | Moisture | Protein | Fat | Ash | Carbohydrate |
|---|---|---|---|---|---|
| Whole flour | 8.21 ±0.11 | 40.33 ±0.16 | 21.92 ±0.24 | 5.12 ±0.01 | 32.63 ±0.46 |
| Defatted flour | 5.69 ±0.09 | 54.17 ±0.48 | 7.13 ±0.64 | 6.28 ±0.09 | 32.42 ±0.46 |
| Protein isolate | 7.94 ±0.21 | 90.75 ±0.28 | 3.67 ±0.12 | 2.32 ±0.07 | 3.26 ±0.17 |

*Note*: Results are expressed as means ± SD for three replications based on a dry weight basis except for the moisture content which is based on a wet basis.

RETRACTED

data using a polynomial quadratic equation which included linear, quadratic, and interaction terms for SPI, TGase, and $CaCl_2$.

Table 2 presents the sequential sum of squares and summary statistics for the models, as well as the results of lack-of-fit (LOF) tests. The appropriate regression models (linear, interactive, quadratic, and cubic) were selected accordingly.

According to the model summaries, the quadratic model presented the best fit for all responses, and the cubic model was found to be aliased. In addition, LOF tests indicated nonsignificant results for the quadratic models for all responses (Table 2). Quadratic models which had greater *R*-squared ($R^2$), adjusted *R*-squared ($Radj^2$), predicted *R*-squared ($Rpred^2$), and smaller standard deviation (Std. Dev.) were chosen for all the cases.

The results of ANOVA for the FCCD design matrix are summarized in Table 3. The selected models were highly significant for all resp[illegible] ($p < .05$) and LOF results were insignificant ($p > .05$). Thus, the mo[illegible]ls are applicable for optimizing the conditions. Table 3 presents $R^2$, a[illegible]justed $R^2$, *p*-value, and the results of LOF tests for all [illegible]dent variables. Model adequacy was evaluated using the L[illegible] test a[illegible] $R^2$. The LOF test failed to produce significant results (*p*[illegible]5), which [illegible]ows that the model fits the empirical data well and [illegible]hat t[illegible] dat[illegible]oints are [illegible] reasonably close to the surface predict[illegible] by the mo[illegible] Small coefficients of variation (CV) attest to the [illegible]recision of the [illegible]periments.

For a model to be adequate, [illegible] $R^2$ val[illegible]arger than 0.8 [illegible]s needed (Wu et al., 2007). In our stud[illegible]$^2$ values for stif[illegible]s, turbidity, syneresis, and loss tangent were 0.9[illegible]0, 0.9949, 0.9954, an[illegible]914, respectively, and $Radj^2$ values were [illegible]9868, 0.99[illegible], 0.9913, and 0.9843, respectively. The negligible differen[illegible] betw[illegible] $R^2$ and $Radj^2$ values reveals the accuracy of the po[illegible]mial mo[illegible]s (Rodrigo[illegible] al., 2012). In addition, adequate pre[illegible]on val[illegible] for all [illegible] res[illegible]nses were greater than 4, which indica[illegible] that the q[illegible]dratic mode[illegible] could navigate the design space.

Th[illegible]nal p[illegible]ions in terms of coded units, after excluding no[illegible]nificant terms, were obtained by RSM as follows: $X_1$ (SPI concentr[illegible]n, %), $X_2$ (TGase, U g$^{-1}$), and $X_3$ ($CaCl_2$, M).

Stiffness = [illegible]00.12 + 87.88$X_1$ + 62.11 $X_2$ + 39.72 $X_3$ + 14.31 $X_1^2$ + 15.95 $X_2^2$-1.61 $X_3^2$ + 40.75 $X_1X_2$ + 24.82 $X_1X_3$ + 13.89 $X_2X_3$.

$$\text{Turbidity} = +0.71 + 0.13X_1 + 0.026\,X_2 + 0.032\,X_3 + 0.0046\,X_1^2 - 0.0038\,X_3^2 \text{-} 0.005\,X_1X_2 - 0.0075\,X_1X_3 - 0.005\,X_2X_3.$$

$$\text{Syneresis} = +0.43 - 0.084X_1 \text{-} 0.006\,X_2 \text{-} 0.034\,X_3 + 0.04\,X_1^2 \text{-} 0.11\,X_2^2 + 0.08\,X_3^2 + 0.0087\,X_1X_3.$$

$$\text{Loss tangent} = +0.62 - 0.23X_1 \text{-} 0.23\,X_2 \text{-} 0.18\,X_3 + 0.0026\,X_1^2 \text{-} 0.0029\,X_2^2 + 0.18\,X_1X_2 - 0.040\,X_1X_3 - 0.020\,X_2X_3.$$

### 3.3 | Gel stiffness

The response surfaces for gel s[illegible]ness are s[illegible]wn in Fi[illegible]re 1a–c. With increasing $CaCl_2$ conce[illegible]ation an[illegible] protein [illegible]t, gel stiffness increased (Figure 1a)[illegible]he effect [illegible] $CaCl_2$ on gel stiffness was more pronounced wh[illegible] highe[illegible]n content [illegible]s used. The highest stiffness of SPI ge[illegible]02.77 g) was [illegible]hieved [illegible]a $CaCl_2$ concentration of 0.6 M, prot[illegible] c[illegible]entrations of [illegible] and TGase concentration of 30 U g$^{-1}$. Supplement[illegible]n with $CaCl_2$ improved the texture of SPI gel and [illegible]ted a more co[illegible]act network due to the formation of ioni[illegible]bonds, which in turn imp[illegible]oved gel strength and texture (And & [illegible]ultin, 2005). A[illegible]ording to Feyzi et al. (2015), SPI has 16.67% gl[illegible]mic acid and 1[illegible]12% aspartic acid on average, which contain two [illegible]boxyl grou[illegible] each. Carboxyl groups can act as binding sites for Ca[illegible] determine gel-forming ability. This effect is likely [illegible]to the involvement of $Ca^{2+}$ ions in electrostatic attractions and [illegible] a res[illegible], reinforcement of the protein network structure. Similar results have been reported for the effect of adding salt to myofibrillar protein gels (Lee et al., 2017) and β-lactoglobulin gels (Mulvihill & Kinsella, 1988). Salt ions increased the strength of protein gels in other cases as well, such as soy protein isolate gel and whey protein gel (Hsia et al., 2016; Otte et al., 1999). Moderate concentration of salt ions induced a denser gel network of arachin–basil seed gum composite gels with higher G′, G″ (G′ > G″) and gel strength via the enhanced electrostatic, hydrophobic, and hydrogen interactions. However, a higher concentration of salt ions by the salting-out effect caused excessive aggregation of arachin, and composite gels were destroyed, which further resulted in a loose structure of the gel with poor hydration properties (Yang et al., 2021). Figure 1b shows the interaction between TGase content and protein concentration. It can be seen that when TGase content increased from 10 to 50 U g$^{-1}$, the stiffness of SPI gel increased. This observation can be explained through the covalent bonding of proteins' lysine and glutamine residues, which enhances texture (Bönisch et al., 2007). At low protein concentrations, TGase caused a nonsignificant change in gel stiffness due to decreased substrate concentration (Ustunol, 2014). Figure 1c presents the variation in stiffness for SPI gel with changes in TGase and $CaCl_2$ concentration at constant protein concentration (10%). The highest stiffness was observed at maximum calcium ion and TGase concentration. Transglutaminase treatment could induce higher gel strength of faba bean protein isolate at neutral pH (Nivala et al., 2021).

**TABLE 2** Adequacy of the model tested (sequential model sum of squares and lack-of-fit tests)

| Sequential model sum of squares | | | | | Lack-of-fit tests | | | |
|---|---|---|---|---|---|---|---|---|
| **Source** | ***df*** | ***F*-value** | ***p*-Value** | **Remark** | ***df*** | ***F*-value** | ***p*-Value** | **Remark** |
| *Stiffness* | | | | | | | | |
| Mean | 1 | | | | | | | |
| Linear | 3 | 30.33 | <.0001** | | 11 | 4.383 E+008 | <.0001** | |
| 2FI | 3 | 25.28 | <.0001** | | 8 | 8.818 E+007 | <.0001** | |
| Quadratic | 3 | 1.752 E+008 | <.0001** | Suggested | 5 | 1.68 | .2905 | Suggested |
| Cubic | 4 | 0.22 | .9159 | Aliased | 1 | 6.68 | [illegible] | Aliased |
| Residual | 6 | | | | | | | |
| Pure error | | | | | 5 | | | |
| Total | 20 | | | | | | | |
| *Turbidity* | | | | | | | | |
| Mean | 1 | | | | | | | |
| Linear | 3 | 1009.30 | <.0001** | | 11 | [illegible] | [illegible] | |
| 2FI | 3 | 24.30 | <.0001** | | 8 | [illegible] | [illegible] | |
| Quadratic | 3 | 3.15 | .0735 | Suggested | 5 | 15.52 | .0049* | Suggested |
| Cubic | 4 | 12.52 | .0045* | Aliased | 1 | 3.62 | .1153 | Aliased |
| Residual | 6 | | | | | | | |
| Pure error | | | | | 5 | | | |
| Total | 20 | | | | | | | |
| *Syneresis (%)* | | | | | | | | |
| Mean | 1 | | | | | | | |
| Linear | 3 | 10.67 | .0004* | | 11 | 318.14 | <.000** | |
| 2FI | 3 | 0.068 | [illegible] | | 8 | 430.67 | <.0001** | |
| Quadratic | 3 | 1510.75 | <.0001* | Suggested | 5 | 0.52 | .7554 | Suggested |
| Cubic | 4 | 0.43 | .7816 | Aliased | 1 | 0.90 | .3872 | Aliased |
| Residual | 6 | | | | | | | |
| Pure error | | | | | 5 | | | |
| Total | 20 | | | | | | | |
| *Loss tangent* | | | | | | | | |
| Mean | 1 | | | | | | | |
| Linear | 3 | [illegible] | <.0001** | | 11 | 37534.95 | <.0001** | |
| 2FI | 3 | 19992.18 | <.0001** | | 8 | 10.56 | .0094 | |
| Quadratic | 3 | 3.76 | .0484 | Suggested | 5 | 7.41 | .0232 | Suggested |
| Cubic | [illegible] | [illegible] | .0079 | Aliased | 1 | 0.45 | .5301 | Aliased |
| Residual | [illegible] | | | | | | | |
| Pure error | | | | | 5 | | | |
| Total | 20 | | | | | | | |

*, **: Significant at [illegible] 5% and $p < 1\%$, respectively.

### 3.4 | Turbidity

The structure of protein gels depends on the nature of the medium, protein concentration, pH, and the type and ionic strength of ions present in the medium (Clark & Lee-Tuffnell, 1998; Gosal & Ross-Murphy, 2000). The influence of protein content, $CaCl_2$, and TGase on the turbidity of SPI gel is illustrated in Figure 2a–c. By increasing both $CaCl_2$ and protein concentration, gel turbidity increased. The effect of protein content on this property was more pronounced compared to that of $CaCl_2$ concentration. Gels with 12% protein had the highest degree of turbidity. The positive effect of $CaCl_2$ concentration on gel turbidity may be due to the formation of structures that are large enough to scatter light (Bryant & McClements, 2000). Furthermore, Kitabatake and Doi reported that as ionic strength increased, insoluble aggregates coagulated and the resulting mass floated in the gel network,

**TABLE 3** Results of ANOVA for response surface quadratic model after optimization results

| | Stiffness | | | Turbidity | | |
|---|---|---|---|---|---|---|
| | *df* | Sum of squares | *p*-Value | *df* | Sum of squares | *p*-Value |
| Model | 9 | 1.547 E+005 | <.0001** | 9 | 0.19 | <.0001** |
| *Linear effects* | | | | | | |
| $X_1$-Protein Concentration | 1 | 77229.49 | <.0001** | 1 | 0.17 | <.0001** |
| $X_2$-$Cacl_2$ Concentration | 1 | 38577.14 | <.0001** | 1 | 6.502 E−003 | <.0001** |
| $X_3$-TGase Concentration | 1 | 15780.36 | <.0001** | 1 | 0.0[illegible] | [illegible]0001** |
| *Quadratic effects* | | | | | | |
| $X_1^2$ | 1 | 563.12 | <.0001** | [illegible] | 5.911 E−0[illegible] | [illegible]203* |
| $X_2^2$ | 1 | 699.42 | <.0001*[illegible] | 1 | 3[illegible]6 E−007 | .8333 |
| $X_3^2$ | 1 | 7.1 | <.0001*[illegible] | 1 | [illegible].105 E−00[illegible] | .0446* |
| *Interaction effects* | | | | | | |
| $X_1X_2$ | 1 | 13287.35 | [illegible]0001** | 1 | [illegible].0[illegible]E−004 | .0005** |
| $X_1X_3$ | 1 | 4926.52 | <.[illegible]1** | 1 | 4.[illegible]00 E−004 | <.0001** |
| $X_2X_3$ | 1 | 1542.48 | <.000[illegible] | 1 | 2.000 E−005 | .0005** |
| Residual | 10 | 6[illegible]43 E−005 | | 10 | 7.794 E−005 | |
| Lack of fit | 5 | [illegible].043 E−005 | [illegible]905 | 5 | 7.310 E−005 | .0049** |
| Pure error | 5 | [illegible].400 E−005 | | | 4.833 E−006 | |
| Cor total | 19 | [illegible]47 E+005 | | 19 | 0.19 | |
| C.V. % | | 0.22[illegible] | | | 0.39 | |
| *R*-Squared | | [illegible].9930 | | | 0.9949 | |
| Adj *R*-Squared | | 0.9[illegible] | | | 0.9904 | |
| *Sy[illegible]* | | | | *Loss tangent* | | |
| [illegible]odel | [illegible] | 0.12 | <.0001** | 9 | 1.65 | <.0001** |
| *Linear ef[illegible]* | | | | | | |
| [illegible]-P[illegible]ein [illegible]ncentration | 1 | 0.071 | <.0001** | 1 | 0.53 | <.0001** |
| $X_2$-Ca[illegible] Conce[illegible]tration | 1 | 3.600 E−004 | <.0001** | 1 | 0.53 | <.0001** |
| $X_3$-TGase Concentration | 1 | 0.012 | <.0001** | 1 | 0.32 | <.0001** |
| *Quadratic effects* | | | | | | |
| $X_1^2$ | 1 | 4.410 E−003 | <.0001** | 1 | 1.911 E−005 | .0260* |
| $X_2^2$ | 1 | 0.033 | <.0001** | 1 | 2.255 E−005 | .0177* |
| $X_3^2$ | 1 | 0.018 | <.0001** | 1 | 3.636 E−007 | .7262 |
| *Interaction effects* | | | | | | |
| $X_1X_2$ | 1 | 1.250 E−005 | .2643 | 1 | 0.26 | <.0001** |
| $X_1X_3$ | 1 | 6.125 E−004 | <.0001** | 1 | 0.013 | <.0001** |
| $X_2X_3$ | 1 | 1.250 E−005 | .2932 | 1 | 3.202 E−003 | <.0001** |
| Residual | 10 | 8.938 E−005 | | 10 | | |
| Lack of fit | 5 | 3.055 E−005 | .7554 | 5 | 2.470 E−005 | .0232 |
| Pure error | 5 | 5.883 E−005 | | 5 | | |
| Cor total | 19 | 0.12 | | 19 | 1.65 | |
| C.V. % | | 0.68 | | | 0.27 | |
| *R*-Squared | | 0.9954 | | | 0.9914 | |
| Adj *R*-Squared | | 0.9913 | | | 0.9843 | |

*Note*: $p < .05$ indicates that model terms are significant.

*, **: Significant at $p < 5\%$ and $p < 1\%$, respectively.

RETRACTED

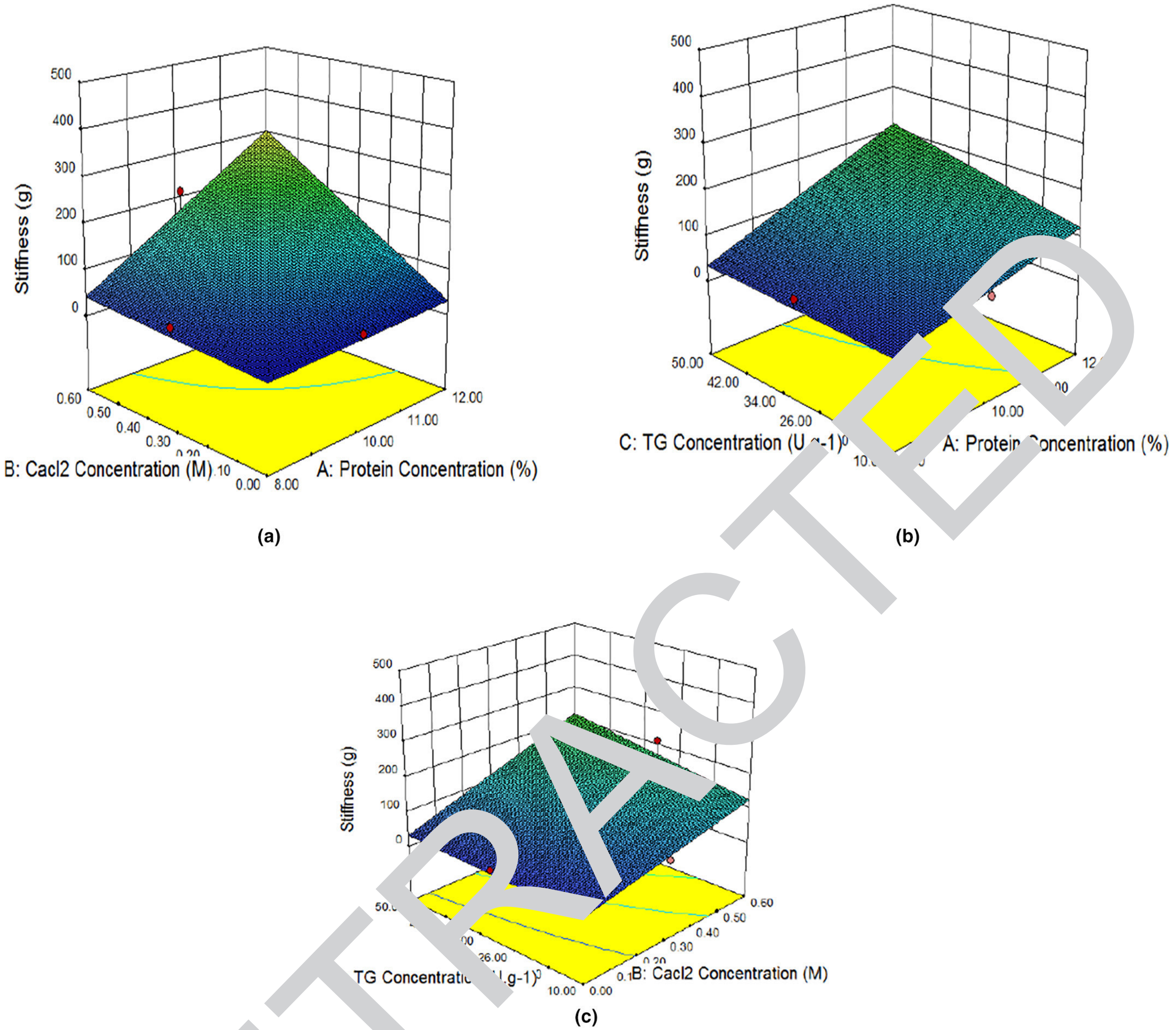

RETRACTED

**FIGURE 1** Response [illegible]rface for the effect of [illegible]Cl$_2$ and protein concentration (a, TG = 30 U g$^{-1}$), TG and protein concentration (b, CaCl$_2$ = 0.30 M), and [illegible]Cl$_2$ and T[illegible]c, protein concentration = 10%) on the stiffness of SPI gel.

leading to turb[illegible]ity in th[illegible]el (Kitaba[illegible]e & Doi, 1993). Similarly, Babaei et [illegible] (2022[illegible]nvestiga[illegible] th[illegible]nfluence of CaCl$_2$ on the turbidity [illegible] whey pr[illegible]ein isolate [illegible]spensions (Babaei et al., 2022). Zhu a[illegible] Damo[illegible] [illegible]ated the influence of CaCl$_2$ concentration [illegible]he turbidity of native whey protein isolate (Zhu & Damodaran, 1[illegible]4) and reported maximum turbidity after treatment with CaCl$_2$ [illegible]0 mM) followed by incubation for 24 hours. They stated that this effect may be associated with salting-in at low and high CaCl$_2$ concentrations and salting-out at 40 mM CaCl$_2$. Conversely, Hongsprabhas and Barbut observed a positive relationship between the opacity of whey protein isolate gels and CaCl$_2$ concentration, and an inverse relationship between the opacity of whey protein isolate gels and protein concentration (Hongsprabhas & Barbut, 1997).

As seen in Figure 2b, when TGase concentration increased from 10 to 50 U g$^{-1}$ and protein concentrations from 8 to 12 U g$^{-1}$ at constant CaCl$_2$ concentration (0.3 M), the turbidity of SPI gel increased to 0.88. The turbidity of proteins is commonly determined by the colloidal characteristics of the proteins and the coagulum formed (Inouye et al., 2002). Hence, the observed increase in the turbidity of SPI gel induced by TGase can be attributed to the formation of insoluble aggregates or coagula. This result is consistent with those reported for the TGase-induced coagulation of soy protein isolate (Tang, Wu, Chen, & Yang, 2006; Tang, Wu, Yu, et al., 2006). In this research, the degree of turbidity varied between 0.5 and 0.88 under various gelation conditions. Minimum turbidity was found at the lowest TGase, CaCl$_2$, and protein concentrations.

### 3.5 | Syneresis

Syneresis is defined as the loss of water during the aging of gels and reflects the instability of the gel network. SPI gels with higher protein concentrations demonstrated less syneresis compared

RETRACTED

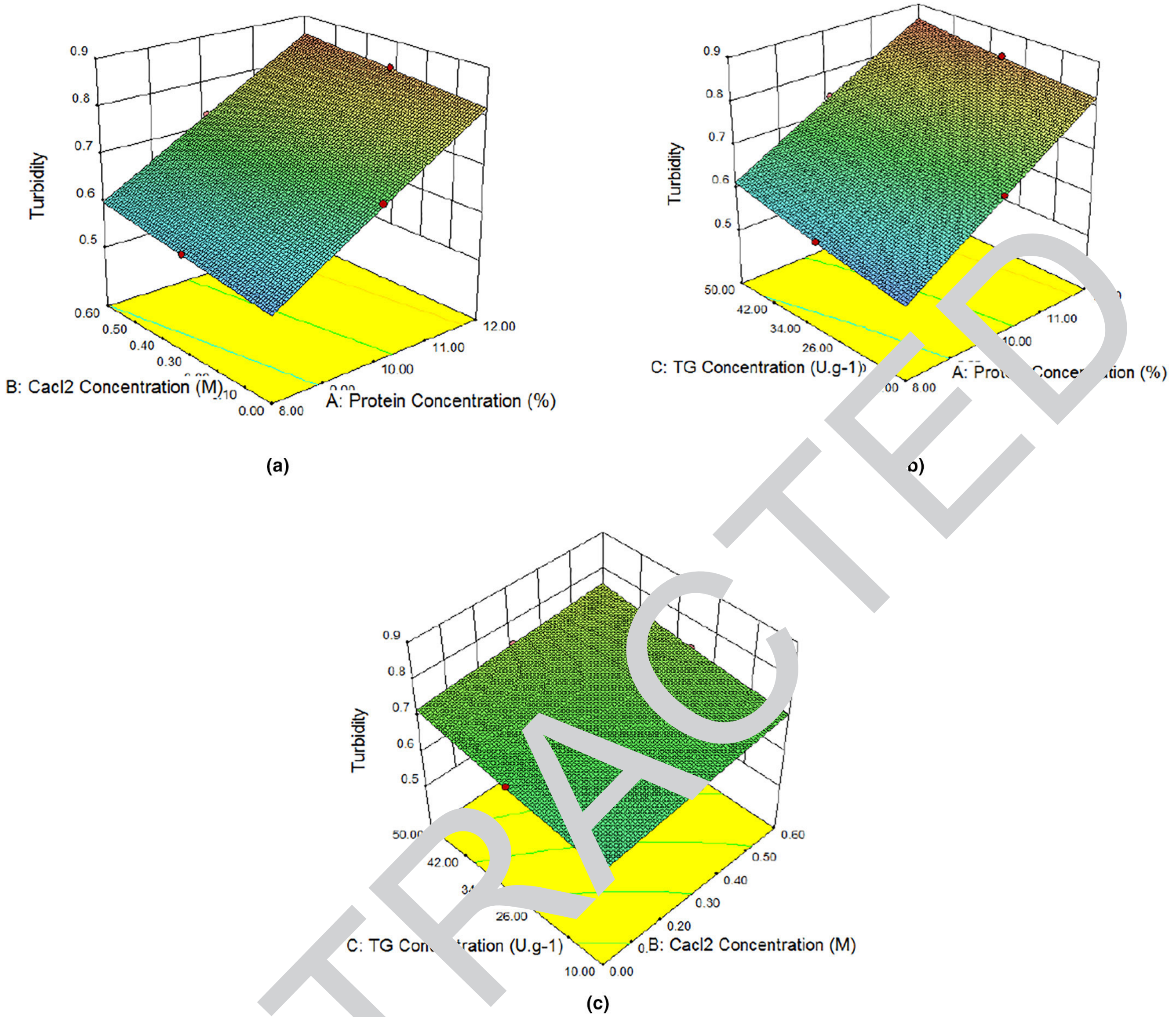

FIGURE 2 Response [illegible]face for the effect o[illegible]Cl$_2$ and protein concentration (a, TG = 30 U g$^{-1}$), TG and protein concentration (b, $CaCl_2$ = 0.30 M), and [illegible]Cl$_2$ and T[illegible](c, protein concentration = 10%) on the turbidity of SPI gel.

to those hav[illegible] lowe[illegible]otein co[illegible]entrations. By increasing protein c[illegible]centr[illegible]on fro[illegible]8% [illegible] 12%, syneresis decreases (Figur[illegible]a) since [illegible]ls with a h[illegible]her protein concentration retain more [illegible]ter du[illegible] [illegible]etwork with greater capillary forces (Remonde[illegible]et al., 2002). It has also been stated that more elastic protein ge[illegible]ave higher water retention capacity (Kinekawa & Kitabatake, 1995[illegible]$CaCl_2$ concentration significantly influences the fundamental structure and mechanical properties of the SPI–WPI gel (Zhang et al., 2022). According to the results of tan $\delta$ measurements, tan $\delta$ decreased and elastic behavior enhanced with increasing protein concentration (Figure 4a), leading to less severe syneresis. By increasing $CaCl_2$ concentration from 0.3 to 0.60 M, syneresis decreased (Figure 3a). The degree of syneresis is determined by gel density and how the gel forms a three-dimensional network (Moritaka et al., 2003). In the presence of $CaCl_2$, the carboxyl groups present in the amino acid structure can act as binding sites for $Ca^{2+}$. The intermolecular ionic interaction between carboxyl groups and $Ca^{2+}$ ions leads to an increase in the shrinkage of the network structure (Moritaka et al., 1995). Therefore, it is expected that the added $CaCl_2$ should reduce syneresis. A similar effect was observed in whey protein and SPI cold-set gels by increasing the concentration of $Na^+$ from 75 to 400 mM and $CaCl_2$ concentration from 10 to 20 mM, respectively (Barbut & Drake, 1997; Maltais et al., 2005), which suggests that the number of available sites interacted with water in the gel also play an important role in retaining water. The number of available sites increases in tandem with the increase in the concentration of monovalent cations, which attracts water molecules from the hydration layer on the surface of aggregated particles, and reduces syneresis (Kuhn et al., 2011). Conversely, the syneresis increased at $CaCl_2$ concentrations below 0.30 M, possibly due to higher porosity (Remondetto et al., 2002; Roff & Foegeding, 1996). Theoretically, water-holding capacity and

RETRACTED

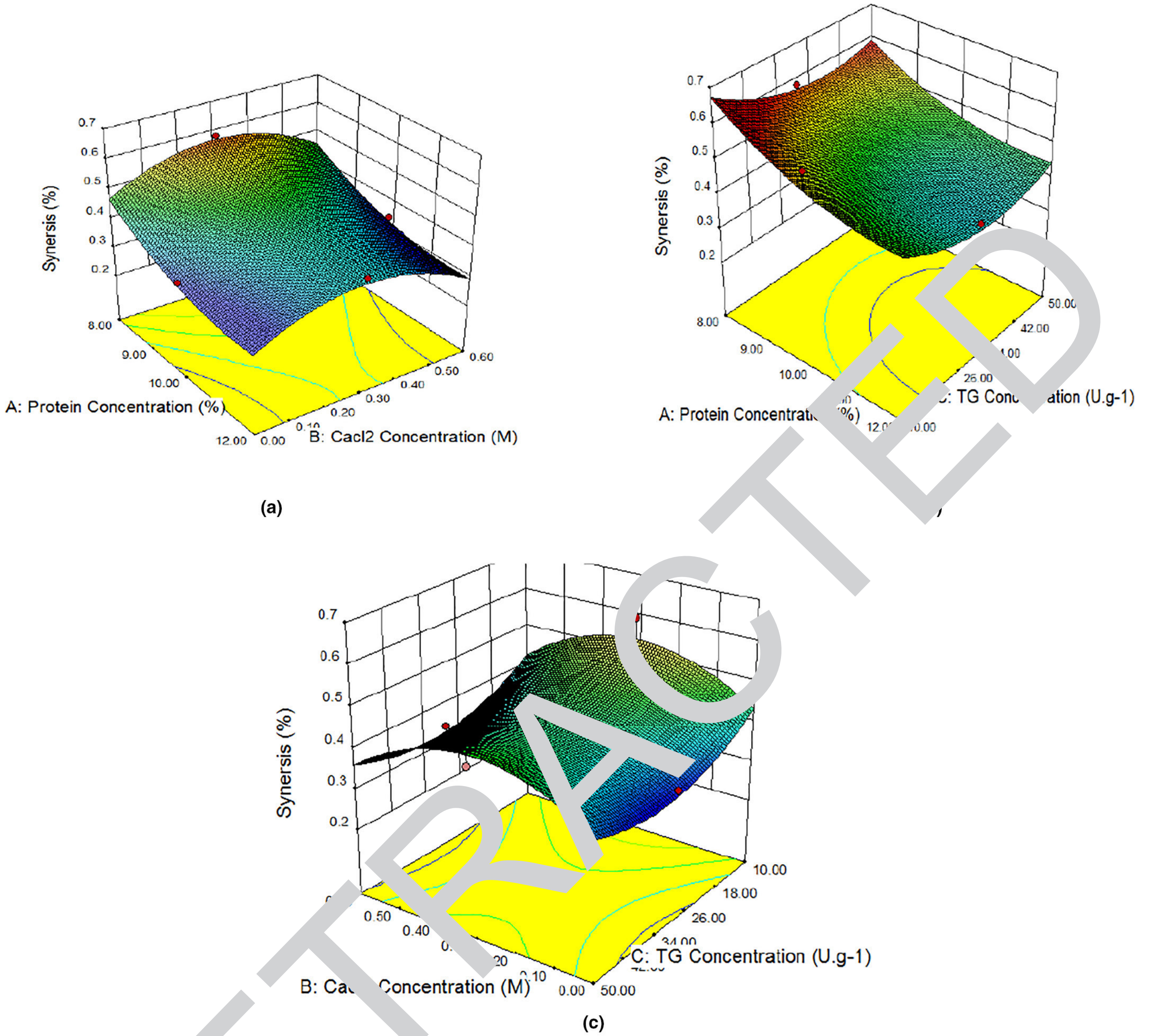

FIGURE 3 Respo[illegible] surface[illegible]r the effect of $CaCl_2$ and protein concentration (a, TG = 30 U $g^{-1}$), TG and protein concentration (b, $CaCl_2$ = 0.30 M), and C[illegible] a[illegible] TG (c, pro[illegible]n concentration = 10%) on the syneresis of SPI gel.

syneresis [illegible] main[illegible] determ[illegible]d b[illegible]orosity and hydrophilic sites in the [illegible] (Chen et [illegible], 2019).

The[illegible]e of T[illegible] [illegible]en to be an appropriate method for altering the[illegible]nnological characteristics of raw products (de Góes-Favoni & Buen[illegible] 2014). For example, this enzyme forms cross-links between protein [illegible]lecules and as a result changes protein properties, gelation ability, water-holding capacity, and thermal stability (Kuraishi et al., 2001). As can be seen in Figure 3b, increasing TGase concentration reduced gel syneresis. This effect can be attributed to the influence of TGase on α-amine groups of lysine residues in proteins, which resulted in an increase in firmness and viscosity, and improved water-holding capacity as a result of reduced syneresis (Kuraishi et al., 2001; Zhu et al., 1995). The proteins cross-linked with transglutaminase showed significantly improved water-holding capacity (5.2–5.6 g/g protein) compared with the control pea protein isolate (2.8 g/g) due to catalyzing covalent cross-linking between lysine and glutamine residues in forming inter- or intra molecular ε-(γ-Glu)-Lys polymers, which results in larger protein molecules and more intensive protein aggregation, favoring water-holding capacity followed by lower syneresis (Shen et al., 2022).

### 3.6 | Loss tangent (tan δ)

Viscoelastic behavior can be described by the loss tangent (tan $\delta$). Loss tangent is calculated by dividing the energy lost in every cycle by the energy stored in every cycle (G″/G′). Fluids are categorized according to their tan $\delta$ as follows: dilute solutions have tan $\delta$ values higher than 1.0, amorphous polymers have tan $\delta$ values between 0.2 and 0.3, and gels and glassy crystalline polymers have tan $\delta$ values near 0.01 (Rao, 1999). According to Figure 4a,b, higher concentrations of proteins resulted in smaller tan $\delta$ values, which can be justified as a manifestation of the

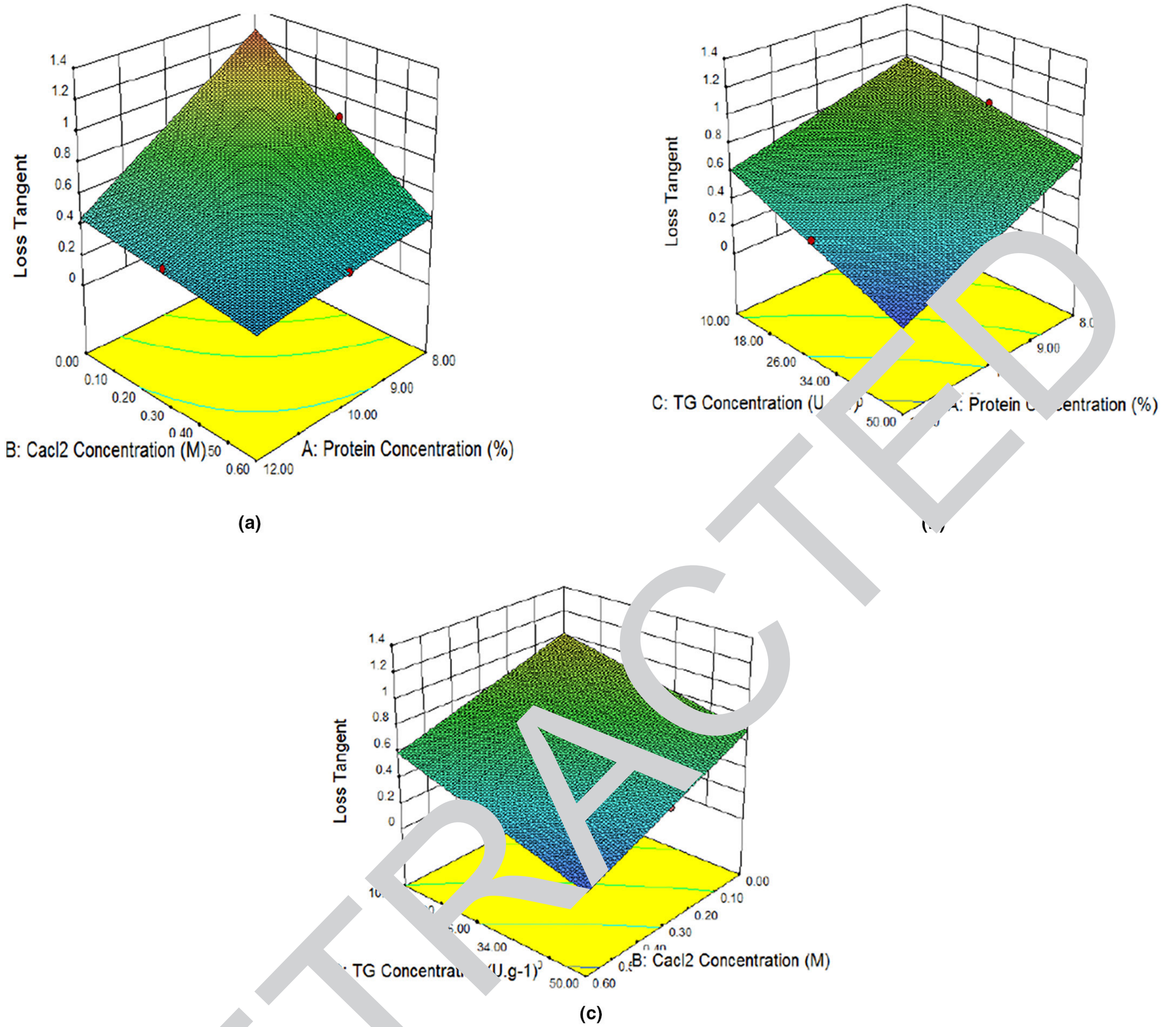

FIGURE 4 Respon[illegible] surface f[illegible] the effect of $CaCl_2$ and protein concentration (a, TG = 30 U $g^{-1}$), TG and protein concentration (b, $CaCl_2$ = 0.30 M), a[illegible] [illegible]a$Cl_2$ an[illegible]G (c, protein concentration = 10%) on tan $\delta$ of SPI gel.

higher co[illegible]lexity [illegible] structu[illegible] fo[illegible]ng at higher protein contents (Ram[illegible] Suárez & [illegible]ong, 2003). [illegible]e same effect was observed in the prese[illegible] of lar[illegible] whey proteins, due to extensive cross-linkage an[illegible] high degree of polymerization (Gauche et al., 2008).

Increasing [illegible]a$Cl_2$ concentration led to a decrease in tan $\delta$ (Figure 4a,c). Th[illegible] [illegible]fluence is likely because of the involvement of $Ca^{2+}$ ions in electrostatic attraction and consequently, improvement of the protein network structure (Ramırez-Suárez & Xiong, 2003).

A similar effect was observed with increasing TGase concentration (Figure 4b,c). The low value of tan $\delta$ in the presence of TGase is due to cross-linking between Gln-Lys and other molecular interactions which result in ordered heteropolymers or aggregates. Such interactions and cross-linking produce gels with great storage moduli (Ramırez-Suárez & Xiong, 2003). This effect was more pronounced in the presence of high amounts of protein, possibly due to the greater number of cross-links, a higher level of polymerization, and greater viscosity (Gauche et al., 2008). With increasing transglutaminase concentration in faba bean isolate protein, tan $\delta$ value reduced to 0.04 which is caused by higher G′ (Nivala et al., 2021).

According to the results, the smallest loss tangent (0.14) was recorded for the sample containing 12% protein, 50 U $g^{-1}$ TGase, and 0.3 M $CaCl_2$, and the highest loss tangent (1.26) belonged to the sample containing 8% protein, 30 U $g^{-1}$ TGase, and 0 M $CaCl_2$ concentration, which indicates that different concentrations of protein, $CaCl_2$, and TGase create a range of different elastic behavior.

#### 3.7 | Optimization of the variables

Optimization was employed to find the best condition for protein gelation. Since the purpose of this study was to apply this protein in different food industries, different conditions were tested. Also, because reducing production costs is an important part of any industry, we tried to minimize enzyme use (due to its relatively high price) and protein content to reduce the cost of the finished product, while maintaining the desired properties of the final gel. The responses including syneresis, tan $\delta$, and turbidity were set to be minimum and the gel's stiffness was set to be maximum.

A weight factor of 1 was selected for all individual desirability values in this design. The most value of 3, displayed by "+++", was chosen for all the responses. Considering these aspects, optimum gelation conditions were evaluated under different conditions (Table 4):

1. When protein content and TGase concentration were set to "is in range" and $CaCl_2$ concentration was set to "is equal to 0".
2. When protein content and $CaCl_2$ concentration were set to "is in range" and $CaCl_2$ concentration was set to "is equal to 0".
3. When all the variables were set to "is in range".
4. When protein content was set to "is in range" and other variables were defined as "minimize".

1. Different levels of protein, TGase, $CaCl_2$, and their combinations create gels with varying stiffness and turbidity. When the mechanical strength and degree of syneresis of the gel are [illegible] most important parameters, we should use the optimized sam[illegible] 3, which is formulated as follows: 10.39% protein, 0.6 M $CaCl_2$ and 41.73 U $g^{-1}$ TGase. On the other hand, when th[illegible]parency of the gel is the most important factor, optimiz[illegible] sample [illegible]should be used. In this case, the optimum conditi[illegible]s for gel [illegible]mation are a protein content of 8.72% and a TGa[illegible]con[illegible]tration [illegible] 10.02 U $g^{-1}$.

**TABLE 4** Optimum operating conditions of SPI gels in different conditions

| | Goal | Lower limit | Upper limit | Optimum |
|---|---|---|---|---|
| *Sample 1* | | | | |
| Protein | Is in range | 8 | 12 | 8.72 |
| $CaCl_2$ | Is equal to 0.00 | 0 | 0.60 | 0 |
| TGase | Is in range | 10 | 5[illegible] | 10 |
| Stiffness | Maximize | 5.08 | 484.05 | [illegible]03 |
| Turbidity | Minimize | 0.5[illegible] | 0.92 | 0.[illegible] |
| Syneresis | Minimize | [illegible].31 | [illegible].68 | 0.5[illegible] |
| Tan (d) | Minimize | 0.17 | 1.[illegible] | [illegible]9 |
| Desirability | | | | 0.59 |
| *Sample 2* | | | | |
| Protein | Is [illegible]ange | 8 | [illegible]2 | 9.06 |
| $CaCL_2$ | [illegible]range | [illegible] | 0.60 | 0.60 |
| TGase | Is equ[illegible] 0.00 | 10 | 50 | 0.00 |
| Stiffnes[illegible] | Maximize | 5.08 | 484.05 | 167.46 |
| Tur[illegible]ity | Minimize | 0.52 | 0.92 | 0.662 |
| S[illegible]eresis | M[illegible]imize | 0.31 | 0.68 | 0.506 |
| T[illegible](d) | Mi[illegible]nize | 0.17 | 1.33 | 0.207 |
| Des[illegible]ility | | | | 0.55 |
| *Sample 3* | | | | |
| [illegible] | Is in range | 8 | 12 | 10.39 |
| $CaCL_2$ | Is in range | 0 | 0.60 | 0.60 |
| TGase | Is in range | 10 | 50 | 41.73 |
| Stiffness | Maximize | 5.08 | 484.05 | 184.33 |
| Turbidity | Minimize | 0.52 | 0.92 | 0.77 |
| Syneresis | Minimize | 0.31 | 0.68 | 0.32 |
| Tan (d) | Minimize | 0.17 | 1.33 | 0.14 |
| Desirability | | | | 0.61 |
| *Sample 4* | | | | |
| Protein | Is in range | 8 | 12 | 9.92 |
| $CaCL_2$ | Minimize | 0 | 0.60 | 0 |
| TGase | Minimize | 10 | 50 | 10.02 |
| Stiffness | Maximize | 5.08 | 484.05 | 81.79 |
| Turbidity | Minimize | 0.52 | 0.92 | 0.57 |
| Syneresis | Minimize | 0.31 | 0.68 | 0.54 |
| Tan (d) | Minimize | 0.17 | 1.33 | 0.19 |
| Desirability | | | | 0.63 |

### 3.8 | Microstructur[illegible]

Cryo-scanning electr[illegible] microsco[illegible] (cryo-SEM) was used to investigate the microstruct[illegible] of t[illegible] gel (Zhou [illegible]et al., 2017). The surface structures of fr[illegible]ze-fract[illegible] SPI gels [illegible]der optimum gelation conditions are [illegible]resent[illegible] in Figu[illegible]5a[illegible] The three-dimensional structure of [illegible]e SPI gel [illegible]ade in the [illegible]esence of both TGase and salt is denser [illegible] mor[illegible]pared to the SPI gel made with TGase or salt alon[illegible]gardless of protein concentration which is confirmed by Zou et al. ([illegible]2), stating more homogeneous and denser three-dimensional netw[illegible] structure of gel using TGase cross-linking of wheat gluten (Zou et al., 2022). Also, adding $CaCl_2$ contributed to the formation of a compact gel phase. $CaCl_2$ promoted protein unfolding and precipitated aggregation by creating Ca links and facilitating hydrophobic interactions. The right concentration of $CaCl_2$ maintains the rate and extent of aggregation required for the formation of an ordered gel network with fewer cavities and improved characteristics, including better strength and syneresis (Arfat & Benjakul, 2012; Tseng et al., 2000; Zhou et al., 2017).

Our findings confirm the importance of $CaCl_2$ concentration in forming a desirable gel. In the presence of TGase, the interaction between Gln-Lys created ordered heteropolymers or aggregates and consequently produced gels with more organized structures (Ramırez-Suárez & Xiong, 2003). This effect was more pronounced in the presence of large amounts of protein, possibly due to the greater number of cross-links, higher level of polymerization, and greater viscosity (Gauche et al., 2008). These findings are in line with those published by Murekatete et al. (2014) and Barbut (1995), who reported that a denser structure was obtained at high ionic strengths. Visualizing the gels' microstructure by cryo-SEM helped to clarify the impact of TGase and $CaCl_2$ on the gels' functional properties.

RETRACTED

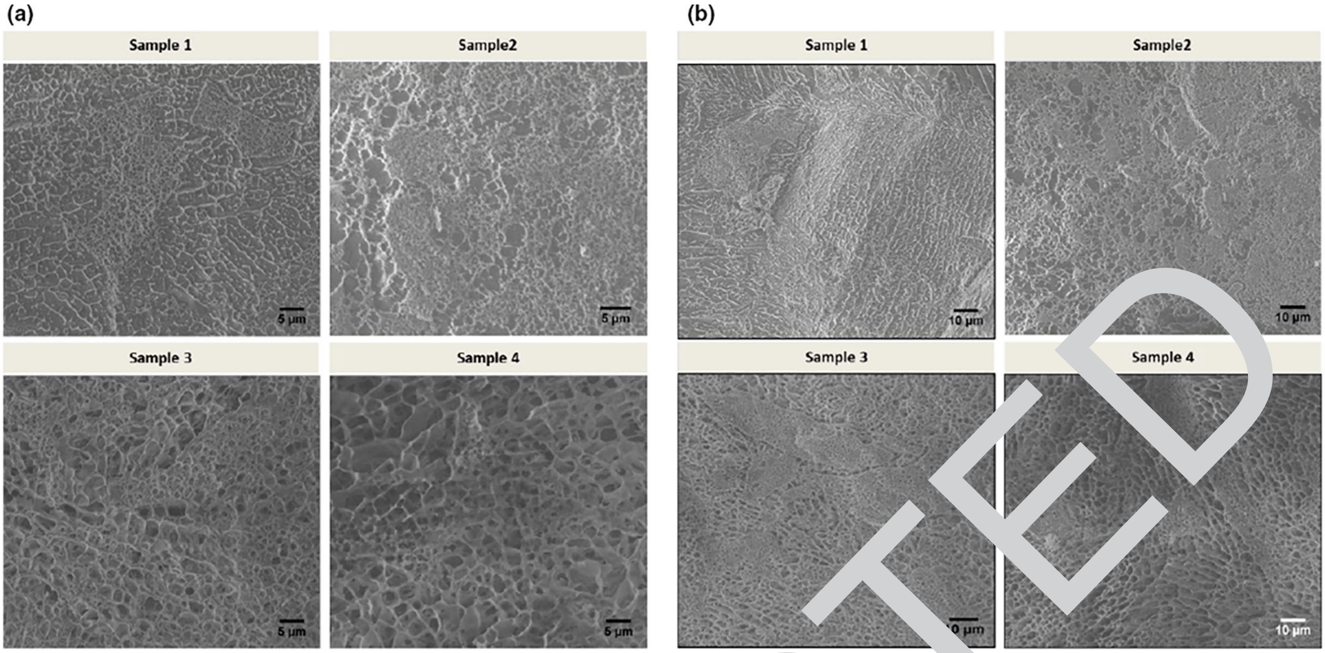

**FIGURE 5** The microstructure of SPI gels observed by cryo-SEM. Sample [illegible] contains 8.72% protein and 10 U $g^{-1}$ TGase. Sample 2 contains 9.06% protein, 0.6 M $CaCl_2$, and no TGase. Sample 3 contains 10.39% prote[illegible], 0.6 M $CaCl_2$, [illegible]d 41.73 U $g^{-1}$ TGase. Sample 4 contains 9.92% protein and 10.02 U $g^{-1}$ TGase. The (a) micrographs show the same areas as [illegible]esented in the ([illegible] micrographs but at greater magnification.

RETRACTED

### 4 | CONCLUSION

In this research, different levels of protein, TGase [illegible]aCl$_2$ produced gels with different levels of strength, syn[illegible]esis, an[illegible] turbidity. Numerical optimization determined the [illegible]timum pr[illegible]paration of soy protein isolate cold-set gels conditions [illegible]sed [illegible] the high[illegible]est stiffness of gels, and the lowest [illegible]neresis, tu[illegible]dity, and loss tangent as being protein content [illegible] 10%, $CaCl_2$ [illegible] 6 M, and TGase concentration of 46 U g[illegible] At this [illegible]timum point, stiffness, turbidity, syneresis, and lo[illegible] tangent were [illegible]nd to be 218.85 (g), 0.76, 0.34 (%), and 0.[illegible]%), respectively. Cry[illegible]EM analysis revealed that the thr[illegible] dimensio[illegible] structures of SPI gels made in the presence of bot[illegible]Gas[illegible]nd $CaCl_2$ were more compact than those formed [illegible]ith TGa[illegible] or salt alo[illegible]. Generally, SPI could be considere[illegible] an eff[illegible]ent ingr[illegible]ent[illegible] make elastic gels. Therefore, new g[illegible]ed foods [illegible]th various t[illegible]xtures and sensory specifications can be [illegible]pand[illegible]

#### ACKNOWLE[illegible]GMENT

The authors grea[illegible] thank Ferdowsi University of Mashhad for financial and laboratory support (grant number: 3/41123).

#### CONFLICT OF INTEREST

All authors have declared that they have no conflict of interest in publishing this research.

#### DATA AVAILABILITY STATEMENT

The datasets generated are available from the corresponding author upon reasonable request.

#### [illegible]ICS STATEMENT

[illegible]is stu[illegible] does not involve any human or animal testing.

#### ORCID

*Mehdi Varidi* https://orcid.org/0000-0003-0885-3975
*Seyed Mohammad Ali Razavi* https://orcid.org/0000-0003-2450-6623
*Farshad Sohbatzadeh* https://orcid.org/0000-0003-3293-0715
